# Gut derived-endotoxin contributes to inflammation in severe ischemic acute kidney injury

**DOI:** 10.1186/s12882-018-1199-4

**Published:** 2019-01-11

**Authors:** Jiangtao Li, Krishna Rekha Moturi, Lirui Wang, Kun Zhang, Chen Yu

**Affiliations:** 1 Department of Nephrology, Tongji Hospital, Tongji University School of Medicine, 389 XinCun Road, Shanghai, 200065 China; 2 Internal Medicine, John H Stroger Jr Hospital of cook county, Chicago, IL 60612 USA

**Keywords:** Acute kidney injury, Renal ischemia reperfusion, Inflammation, Intestinal permeability, Endotoxin

## Abstract

**Background:**

Recent researches indicate that the intestinal consequences of renal ischemia reperfusion (IR) would predispose to the translocation of gut-derived endotoxin. Here, we designed experiments to test the hypothesis that the gut-derived endotoxin has a potential role in mediating local inflammatory processes in the acutely injured kidney.

**Methods:**

Rats were performed sham or renal IR surgery (60 min of bilateral renal ischemia, then 24 h of reperfusion) (*n* = 5). The intestinal structural and mucosa permeability were evaluated. Serum endotoxin and bacterial load in liver and mesenteric lymph nodes (MLN) were measured. Separate groups were pretreated with oral norfloxacin 20 mg/kg/day or saline for 4 weeks and divided into sham plus saline, sham plus norfloxacin, renal IR plus saline and renal IR plus norfloxacin group. Serum biochemistry and endotoxin were determined. Kidney pathological changes were scored. Protein or mRNA expression of toll-like receptor 4 (TLR4) and proinflammatory mediators were measured in kidney homogenate.

**Results:**

Renal IR led to marked intestinal integrity disruption and increase in intestinal permeability. These are accompanied by low grade of endotoxemia as well as increased bacterial load in liver and MLN. The group pretreated with norfloxacin showed significant attenuation of the increase in serum urea, ALAT, ASAT and endotoxin. The increased renal protein or mRNA of TLR4 and proinflammatory mediators (IL-6 and MCP-1) in the unpretreated animals was significantly attenuated in the norfloxacin-pretreated animals. However, norfloxacin pretreatment did not produce any protective effects on renal tubular integrity.

**Conclusions:**

Our results show for the first time that gut-derived endotoxin, resulting from an increased intestinal permeability after severe renal IR, subsequently amplifies intrarenal inflammatory response by activation renal TLR4 signaling. Our study results do not establish that antibiotic administration was effective in improving the overall renal outcome. However, our findings may be the first step to understanding how to tailor therapies to mitigate intrarenal inflammation in select groups of patients.

**Electronic supplementary material:**

The online version of this article (10.1186/s12882-018-1199-4) contains supplementary material, which is available to authorized users.

## Background

Acute kidney injury (AKI) is a serious complication with no special therapies currently. Renal ischemia-reperfusion (IR) is one of the major causes of AKI [1]. The pathophysiology of IR-induced renal injury includes oxidative stress, inflammation and damage to the vascular endothelium and tubular epithelium [2]. Among them, inflammation is possibly the most important pathophysiological factor involved in the propagation of renal IR.

The gut bears critical immunologic and barrier functions that prevent the passage of substances, such as pathogenic microorganisms and their products, antigens and proinflammatory mediators from the intestinal lumen into the internal milieu. The disruption of gut barrier is proposed to permit the translocation of intestine endotoxin/bacteria into the portal vein and peripheral circulation where host defenses are already compromised, leading to aggravation of the inflammatory status and propagation of inflammation [3]. A rather new research using renal IR mice demonstrated a series of intestinal consequence of ischemic AKI, manifested by the increased intestinal permeability, enhanced inflammation, profound apoptosis and necrosis [4]. However, whether the consequences of renal IR would predispose to the translocation of endotoxin and/or bacterial components is not clear.

Toll-like receptors (TLRs) are key members of the innate immune system, serving as pattern recognition receptors that identify a wide spectrum of microbial products. TLR4 is one of the key TLRs which critically involved in inflammation [5, 6]. TLR4 is expressed by cells of the immune system, such as dendritic cells, macrophages, neutrophils, B cells and NK cells. TLR4 can also be expressed in tissue by cell types, including kidney mesangial cells and tubular epithelial cells in response to injury [7]. Activation of TLR4 by LPS generates an excessive production of inflammatory mediator [7, 8]. During the process of renal IR, whether the gut-derived endotoxin would further contribute to renal inflammation through the TLR4 mediated pathway remains to be clarified.

In present study, we used a rat model of severe renal IR and examined (1) whether the translocation of endotoxin and/or bacterial components would occur as the intestinal consequences of renal IR and (2) whether the gut-derived endotoxin would further contribute to renal inflammation through TLR4 activation.

## Materials and methods

### Ethical approval and animals

The animal use and care protocol and experimental procedures were reviewed and approved by the Experiment Animal Center Committee in Tongji University and the procedures were carried out in accordance to the National Institute of Health Guidelines for the Care and Use of Laboratory Animals (8th edition; available online: https://www.ncbi.nlm.nih.gov/books/NBK54050/) and to the European Community Council Directive for the Care and Use of Laboratory Animals (Directive 2010/63/EU; http://ec.europa.eu/environment/chemicals/lab_animals/legislation_en.htm). Sprague-Dawley male rats (Shanghai SLAC Laboratory Animal Co., Ltd., Shanghai, PR China), weighing 200-250 g, were bred at a stable temperature of 22–23 °C with 12-h light-dark cycles. They were permitted to get access to diet and water freely. All procedures were performed using sterile techniques under adequate anesthesia.

### Experimental protocol

The first set of experiments was performed to test the hypothesis that the intestinal consequences of renal IR would predispose to the translocation of endotoxin and/or bacterial components. To do so, a well-characterized rat model of AKI induced by severe renal IR was used. Prior to surgeries, all rats were randomly allocated into sham operation group and renal IR group (*n* = 5 for each group). Intestinal structural alterations and permeability were assessed. Translocation of gut-derived endotoxin and bacteria was detected.

The second group of experiments was conducted to study whether the gut-derived endotoxin would further contribute to renal inflammation during renal IR. By doing so, norfloxacin was used to reduce the translocation of gut-derived endotoxin through inhibiting intestinal flora overgrowth. Briefly, the rats were randomly pretreated with oral norfloxacin (Sigma-Aldrich, USA) 20 mg/kg/day or saline for 4 weeks and then divided into 4 groups (*n* = 5 for each group), including sham plus saline, sham plus norfloxacin, renal IR plus saline and renal IR plus norfloxacin. On any given day, the person administering norfloxacin or saline was blinded to treatment. The effects of norfloxacin on endotoxinemia, renal and hepatic dysfunction, pathological changes and inflammatory response in kidney were investigated.

Since our study is a pilot study and prior information is not available, sample size was not determined by power analysis. However, five animals per group for continuous endpoints is reasonable [9]. Animals randomization in our study was conducted by Excel software (Excel 2003, Microsoft Corporation, One Microsoft Way, Redmond, WA, USA).

### Renal IR

Thirty  min after intraperitoneal injection of 2% pentobarbital sodium (50 mg/kg), the rats were fully anesthetized and then fixed on a heating plate to keep a rectal temperature around 37 °C. The renal pedicles were isolated after a midline laparotomy. For renal IR induction, vascular clamps were placed around both renal pedicles for 60 min, followed by 24 h of reperfusion. After clamps removal, kidneys were inspected for their original color recovery. To avoid excessive fluids loss, rats were administrated by 1 mL warm sterile saline intraperitoneally prior to abdomen closing. For sham animals, identical surgical procedures were conducted except the induction of IR.

### Collection and preparation of samples

With overdose of 2% pentobarbital sodium, rats were euthanized 24 h after surgery. Blood was collected by intracardic puncture, centrifuged (4000 g for 10 min at 4 °C) and the serum was stored at − 70 °C for further measurements. Samples from kidney, liver and mesenteric lymph nodes (MLN) were snap-frozen in liquid nitrogen and stored at − 70 °C for further use. Tissues of kidney and ileum were fixed in formalin (10% phosphate-buffered, pH = 7.4) for histological evaluations.

### Biochemistry

Serum levels of creatinine, urea, alanine aminotransferase (ALAT) and aspartate aminotransferase (ASAT) were determined by standard methods on an Olympus AU 2700 Analyzer (Olympus Optical Co., Ltd., Tokyo, Japan). All determinations were performed blindly with respect to group allocation or treatment.

### Serum D-lactate concentration

A serum D-lactate quantitative colorimetric detection kit (Genmed, Boston, United States) was used to determine serum D-lactate levels. Results were expressed as mmol/L. All determinations were performed blindly with respect to group allocation.

### Protein levels of cytokines in kidney tissue

The sample of kidney was homogenized and the supernatants were subjected to protein concentration determinations using bicinchoninic acid (BCA) method (Nan jing jian cheng bioengineering institute, Nanjing, China). Quantitative assessment of tumor necrosis factor (TNF-α), interleukin-6 (IL-6) and monocyte chemotactic protein-1 (MCP-1) protein levels were measured by commercial available ELISA kits (MultiSciences, Hangzhou, China). Results were expressed as pg/mg protein. All determinations were performed blindly with respect to treatment.

### Measurement of endotoxemia

Endotoxin quantification was determined by a Kinetic Turbidimetric LAL Kit (Xiamen Limulus Experimental Reagents Factory, Xiamen, China). Results were expressed as EU/mL. All determinations were performed blindly with respect to group allocation or treatment.

### In vitro intestinal permeability

After 24 h surgery, length of 5 cm terminal ileum segment was excised. Ileum lumen was gently flushed, and one end of ileum was fastened by ligation. Next, 200ul of 40 mg/mL FITC-Dextran (MW, 4400 Da, FD-4) (Sigma-Aldrich, USA) was poured into ileum lumen and another end was fastened. The ileum sac was vibrated carefully in 20 mL of saline under 37 °C for 60 min. Permeability of ileum wall was assessed in vitro by measuring the amount of FITC-dextran in saline [10]. All measurements were performed blindly with respect to group allocation.

### Bacterial load quantification

Bacterial DNA load was measured in liver and MLN. Total DNA was extracted from tissues homogenates by DNEASY Blood & Tissue Kit (Qiagen, Germany). The primer sets applied (Table 1) in present study targeted a conserved region of the 16srRNA gene present in universal bacteria. The methods for bacterial DNA amplification has been described by Balamurugan R [11]. To normalize the variable mass of the collected tissue samples, primers for the β-actin gene were used. Bacterial 16srRNA gene expression was normalized to β-actin in each sample and calculated by the 2^−ΔΔCt^ method. All PCR analysis were performed blindly with respect to group allocation.

**Table 1:** List of primers used to quantify bacteria

Gene	Forward Primer	Reverse Primer
β-actin	5’-TCGTACCACTGGCATTGTGATGGA-3′	5’-ACCGCTCATTGCCGATAGTGATGA-3′
16 s rRNA	5’-TCCTACGGGAGGCAGCAGT-3′	5’-GGACTACCAGGGTATCTAATCCTGTT-3′

### mRNA levels of cytokines in kidney

Total RNA was extracted by TRI reagent (Sigma-Aldrich, USA) according to the instruction from the manufacturer. Complementary DNA (cDNA) was synthesized by using a commercial cDNA synthesis kit (Thermo Scientific, USA). mRNA expression of TNF-a, IL-6 and MCP-1 was measured by real-time quantitative RT-PCR conducted on a Light Cycler 480 (Roche) with SYBR green PCR master mix (Taraka, USA). Expression of targeted genes was normalized to β-actin expression in each sample and calculated using the 2^−ΔΔCt^ method. Primer sequences are listed in Table 2. All RT-PCR analysis were performed blindly with respect to treatment.

**Table 2:** Oligonucleotide primer sets for real-time PCR

Gene	Forward Primer	Reverse Primer
β-actin	5’-AGATTACTGCCCTGGCTCCTAG-3’	5’-CATCGTACTCCTGCTTGCTGA-3’
TNF-α	5’-TGCCTCAGCCTCTTCTCATT-3’	5’-GGGCTTGTCACTCGAGTTTT-3’
IL-6	5’-AGTTGCCTTCTTGGGACTGA-3’	5’-ACAGTGCATCATCGCTGTTC-3’
MCP-1	5’-GATGCAGTTAATGCCCCACT-3’	5’-TTCCTTATTGGGGTCAGCAC-3’

### Western blot

Total proteins from kidney were separated by SDS-PAGE and transferred to nitrocellulose membranes (Bio-Rad). Blots were probed with primary specific antibodies followed by horseradish peroxidase-conjugated goat anti-rabbit IgG antibody (ab97051; Abcam, 1:10000). The immunoreactive bands densities were quantified using NIH Image J software (version 1.47). Primary antibodies were the following: anti-TLR4 (ab22048; Abcam, 1:500) and anti-GAPDH (ab37168; Abcam, 1:1000). All western blot analysis were conducted blindly with respect to treatment.

### Histology

After formalin fixation and dehydration, paraffin-embedded tissue sections (4 μm) were stained by hematoxylin and eosin (HE). The histopathological changes were evaluated under a light microscope, independently by two pathologists blinded to the present study design. Renal tubular necrosis was assessed using semi-quantitative score. Renal necrosis was defined as: 0: 0%, 1: 1–10%, 2: 11–25%, 3: 26–50%, 4: 51–75% and 5: more than 75% of tubules are necrotic. The histopathological changes of ileum were evaluated by Chiu’s scoring system [12]. Chiu’s score grading was as follows: Grade 0, normal mucosal villi; Grade 1, development of subepithelial Gruenhagen’s space, usually observed at the apex of the villus, often with capillary congestion; Grade 2, extension of the subepithelial Gruenhagen’s space with moderate lifting of epithelial layer from the lamina propria; Grade 3, massive epithelial lifting down the sides of villi, with a few tips being denuded; Grade 4, denuded villi with lamina propria and dilated capillaries exposed, increased cellularity of lamina propria; and Grade 5, digestion and disintegration of lamina propria, hemorrhage, and ulceration.

### Immunohistochemistry (IHC)

IHC was performed on paraffin-embedded sections of kidney specimens. TLR4 was detected with anti-TLR4 (ab22048; Abcam, 1:250) and then incubated with horseradish peroxidase-conjugated goat anti-rabbit IgG H&L antibody (ab97051; Abcam, 1:150). The samples were stained with a diaminobenzidine staining kit (DAKO). All IHC experiments were carried out blindly with respect to treatment.

### Statistical analyses

Graphpad Prism 4.0 (GraphPad Software, Inc., San Diego, USA) was used for statistic analysis. Differences between two groups were compared by two-tailed unpaired t test. Comparison among multiple groups was analyzed with one-way ANOVA followed by Bonferroni multiple comparison method. The data were presented as mean ± SD. Values of *P* < 0.05 were considered as statistically significant.

## Results

### Intestinal mucosal injury after renal IR

**Fig. 1:**
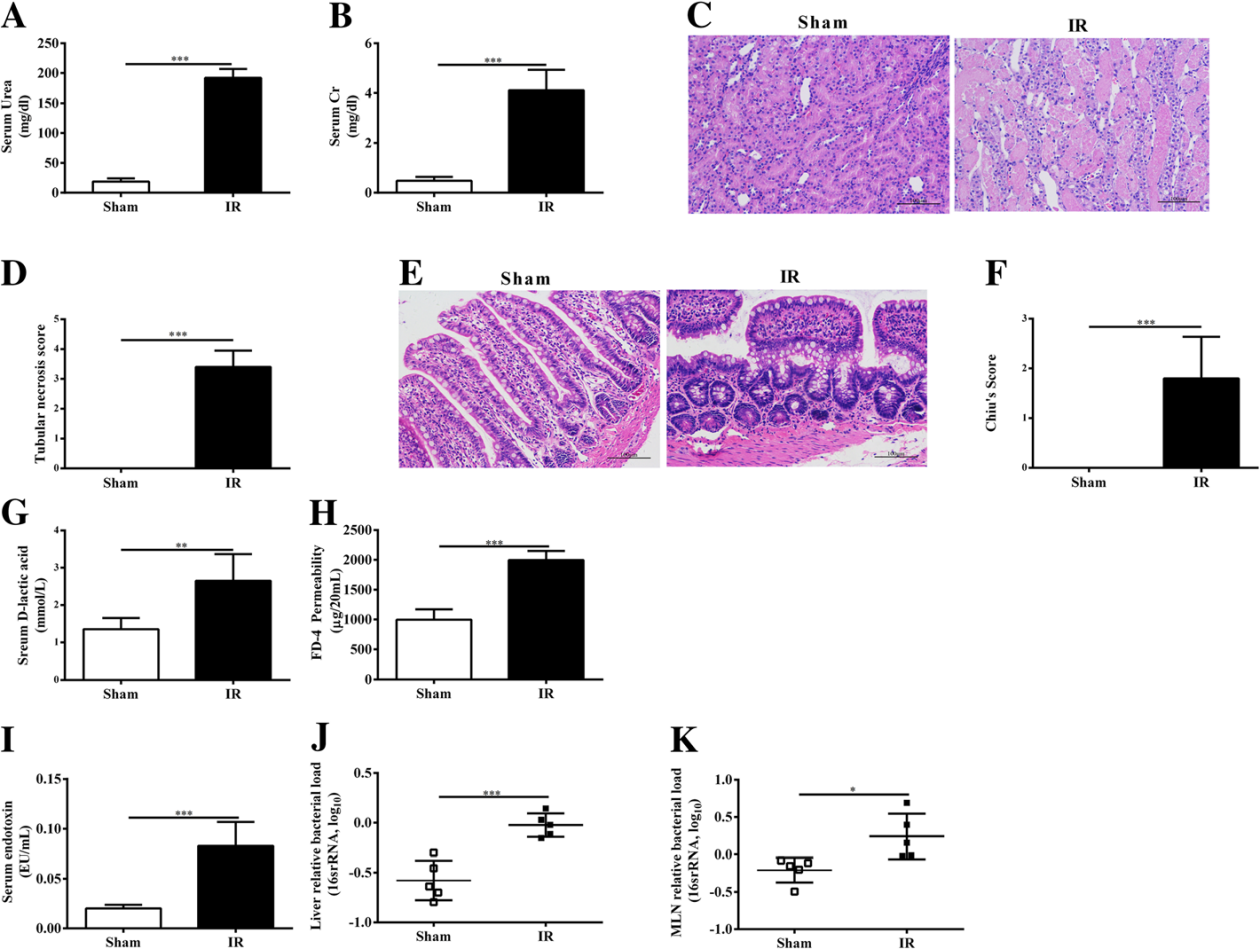
The intestinal consequences of renal IR induced AKI. Renal function was evaluated by serum urea (**a**) and creatinine (**b**) levels. Kidney and ileum morphological alterations were evaluated by HE stained sections (original magnification× 200) and scored (**c**-**f**). Intestinal permeability was evaluated by serum D-lactic (**g**) and an ex vivo isolated sac method (**h**). The level of serum endotoxin was measured using a Kinetic Turbidimetric LAL method (**i**). Bacterial load was measured at liver (**j**) and mesenteric lymph nodes (**k**). Bacterial load was represented by relative bacterial load in log as quantified by qPCR of 16S primer targets normalized to β-actin. Higher values represent more bacteria. Data are expressed by mean ± SD. The two-tailed unpaired t test was used (*n* = 5 per group). *P < 0.05, **P < 0.01, ***P < 0.001

Renal IR led to a pronounced kidney injury, evidenced by a markedly rise in serum urea and creatinine as compared to the sham rats (*P* < 0.001) (Fig. 1a, b). This was associated with evidence of renal morphological damage as reflected by increased necrotic tubules in the renal IR rats compared to the sham rats (Fig. 1c, d).

The gut wall forms an anatomical barrier and the breakdown of this barrier potentially leads to the translocation of microbiota and their products. Therefore, we examined the morphological alterations in ileum. The integrity of ileum mucosa under light microscopy was nearly normal in the sham rats. By contrast, edema and inflammatory cells were observed in the ileal mucosa and submucosa in renal IR rats. The difference in the degree of ileal mucosal injury between groups were supported by the results of Chiu’s scoring, in which renal IR rats presented higher score than the sham rats. (Fig. 1e, f). Taken together, these data clearly show that renal IR results in a pronounced breakdown of intestinal physical barrier.

### Increased intestinal mucosal permeability after renal IR

We next evaluated the impact of renal IR on mucosal permeability by two separate methods. The serum level of the bacterial fermentation product D-lactate has been used as an effective marker to evaluate intestinal mucosal permeability. Bacterial overgrowth and the disruption of the mucosal barrier, which result in the leakage of bacterial metabolic products into the circulation, are attributed to the elevation of serum D-lactate level. In our study, there was a markedly elevation in the serum D-lactate levels detected in the rats subjected to renal IR, when compared with the sham rats (*P* < 0.01) (Fig. 1g). The permeability of ileum wall was further assessed by using the ex vivo isolated sac method. In the renal IR rats, the amount of FD-4 that passed the wall of ileum was significantly higher than that of the sham rats (*P* < 0.001) (Fig. 1h). Together these findings show that renal IR leads to an increased intestinal mucosal permeability.

### Renal IR leads to endotoxinemia and bacterial translocation

Breakdown of the intestinal barrier potentially leads to the translocation of microbiota and their products. Endotoxin levels in peripheral blood from rats 24 h after renal IR were significantly higher than that of the sham rats (*P* < 0.001) (Fig. 1i). The traditional culture and colony counting method is insufficient to detect all species of microbiota. Therefore, a more sensitive approach using qPCR by targeting bacterial conserved 16srRNA sequences was employed to quantify bacterial load in liver and MLN. We found that the renal IR rats had significantly greater bacterial load in liver and MLN, when compared with the sham rats (liver: *P* < 0.001; MLN: *P* < 0.05) (Fig. 1j, k). Overall, these findings show that the disruption of intestinal barrier in renal IR allows the translocation of bacteria and endotoxin.

### Norfloxacin pretreatment shows no protective effects on renal outcome

**Fig. 2:**
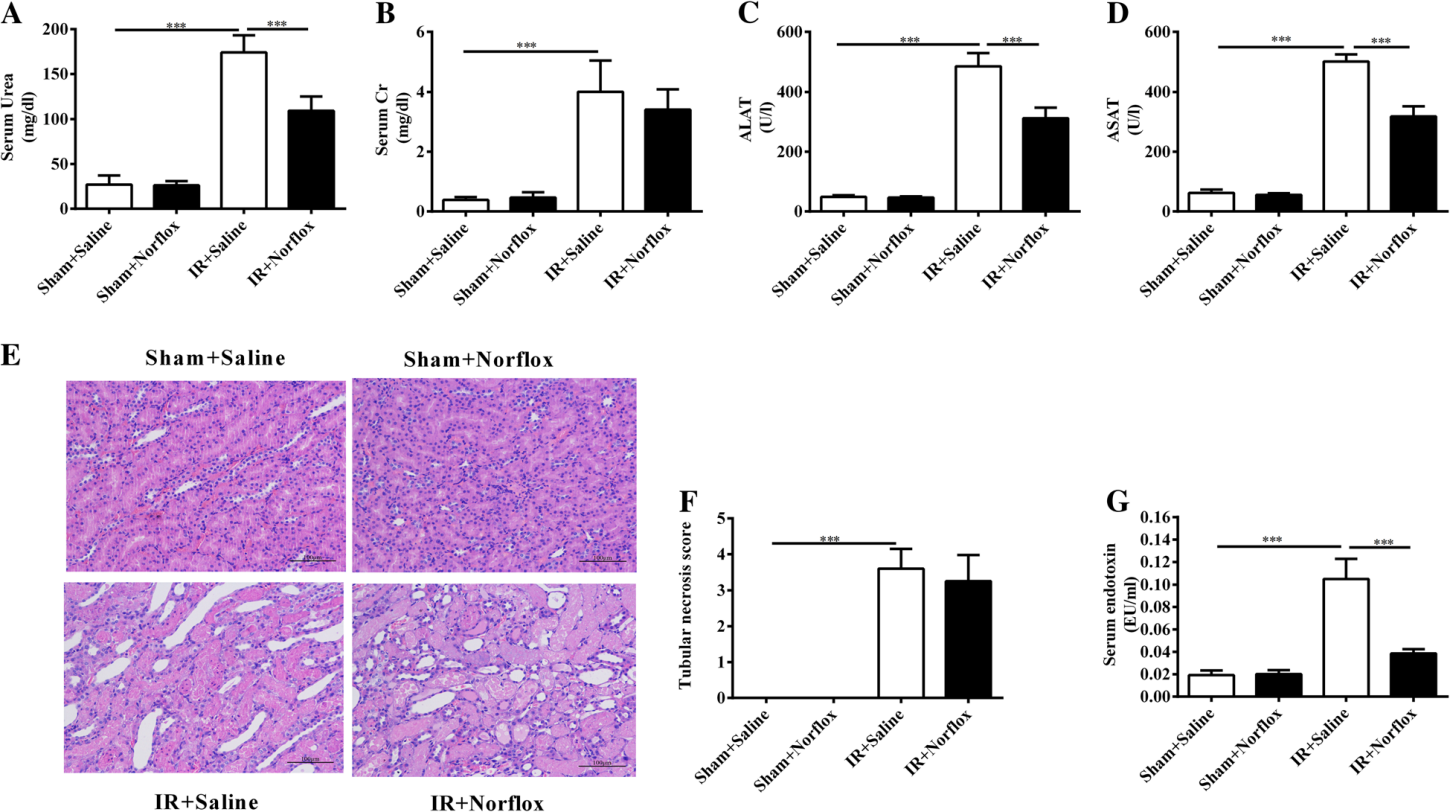
The effects of norfloxacin pretreatment on renal and liver injury. Renal function was evaluated by serum urea (**a**) and creatinine (**b**) levels. Liver function evaluated by serum ALAT (**c**) and ASAT (**d**) were determined. Tubules necrosis was scored in HE stained sections (original magnification× 200) (**e**, **f**). Serum endotoxin levels measured using a Kinetic Turbidimetric LAL method (**g**). Data are presented as mean ± SD. One-way ANOVA followed by Bonferroni multiple comparison test was used in all graphs (*n* = 5 per group). ***P < 0.001

Renal IR led to an increase in serum urea and creatinine (*P* < 0.001, respectively), along with tubular necrosis in renal tissues (*P* < 0.001). Norfloxacin pretreatment reduced the serum urea levels in renal IR rats (renal plus norfloxacin) (*P* < 0.001) (Fig. 2a). However, no differences were observed with respect to serum creatinine (Fig. 2b) and tubular necrosis scores (Fig. 2e, f) between the renal IR rats (renal IR plus saline) and renal IR rats with norfloxacin pretreatment (renal IR plus norfloxacin). Taken together, these data suggest that norfloxacin pretreatment has no substantial benefits to renal outcome.

### Norfloxacin pretreatment protests against liver dysfunction following renal IR

The ALAT and ASAT values were markedly elevated in the renal IR rats (renal IR plus saline) compared with the sham rats (sham plus saline) (*P* < 0.001, respectively). Norfloxacin pretreatment reduced the severity of liver dysfunction in renal IR rats (renal IR plus norfloxacin), as shown by a reduction in the serum ALAT and ASAT values (*P* < 0.001, respectively) (Fig. 2c, d). In conclusion, these data indicate that norfloxacin pretreatment protects liver dysfunction.

### Norfloxacin pretreatment attenuates renal IR-induced endotoxinemia

As expected, serum endotoxin levels were significantly higher in the renal IR rats (renal IR plus saline) compared with the sham rats (sham plus saline) (*P* < 0.001). By contrast, pretreatment of the renal IR rats with norfloxacin (renal IR plus norfloxacin) significantly reduced their serum endotoxin levels (*P* < 0.001) (Fig. 2g). In summary, these data show that four-week oral norfloxacin pretreatment is effective on attenuating renal IR-induced endotoxinemia.

### Norfloxacin pretreatment reduces renal TLR4 protein expression

**Fig. 3:**
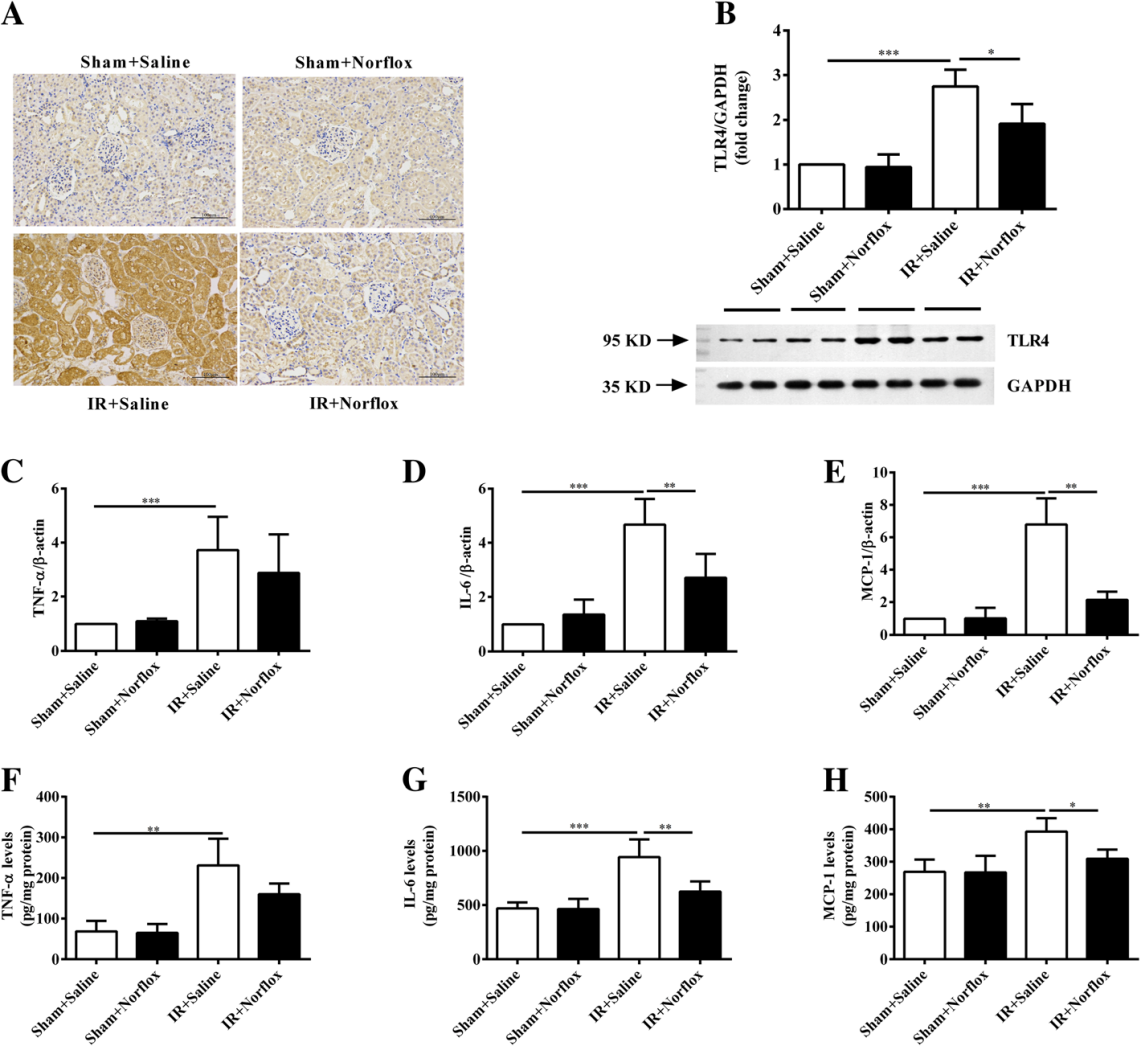
Norfloxacin pretreatment reduces kidney TLR4 protein expression and renal proinflammatory mediators production. (**a**) Immunohistological staining of TLR4 in the kidney. (**b**) TLR4/GAPDH protein expression on densitometry. mRNA and protein levels of TNF-α (**c**, **f**), IL-6 (**d**, **g**) and MCP-1 (**e**, **h**) were measured in kidney hemogenates. Data are presented as mean ± SD. One-way ANOVA followed by Bonferroni multiple comparison test was used in all graphs (n = 5 per group). *P < 0.05, **P < 0.01, ***P < 0.001. (Original blots ‘BMC Additional file 1)

To further examine whether pretreatment with norfloxacin modulates renal TLR4 expression, we performed IHC and Western blot. IHC revealed that renal IR led to an increase expression of renal TLR4 protein. Pretreatment of the renal IR rats with norfloxacin (renal IR plus norfloxacin) reduced their renal expression of TLR4 (Fig. 3a). These findings were further confirmed by Western blot (Fig. 3b). Taken together, these results show that renal TLR4 expression is increased in renal IR rats and its expression can be modulated by norfloxacin pretreatment.

### Norfloxacin pretreatment decreases renal proinflammatory mediators

Proinflammatory cytokines and chemokines contribute to the pathophysiology of renal IR. To further evaluate the effect of norfloxacin pretreatment on renal inflammation, both mRNA and protein levels of TNF-a, IL-6 and MCP-1 in kidney homogenates were detected. The mRNA levels of TNF-a, IL-6 and MCP-1 were markedly increased in the renal IR rats (renal IR plus saline) compared with the sham rats (sham plus saline) (all *P* < 0.001). Pretreatment of the renal IR rats with norfloxacin (renal IR plus norfloxacin) significantly reduced mRNA levels of these proinflammatory mediators in kidney tissue (IL-6: *P* < 0.01; MCP-1: *P* < 0.01) (Fig. 3c-e). Furthermore, the protein levels of these proinflammatory mediators assessed by ELISA were consistent with the mRNA results (Fig. 3f-h). These findings indicate that norfloxacin pretreatment could abrogate the renal inflammatory signaling cascade.

## Discussion

AKI induced-gut injury was first reported in 2011. Over the past years, however, the clinical importance of intestinal consequences during AKI went somewhat out of the focus of nephrologists. Here, we showed that gut-derived bacterial endotoxin, resulting from the disrupted gut barrier due to renal IR, evokes a TLR4 mediated inflammatory response in the kidney. Our study provides new insights into the mechanism of inflammation in ischemic AKI.

In a renal IR model of rats, Mehri and colleagues reported that a minimum of 45 min ischemia is required to study the impacts of renal injury on distant organs [13]. On the basis of their observations, we selected a renal IR model of 60 min ischemia to address our hypothesis. As expected, severe renal injury resulted in striking morphological alterations in ileum. On the other hand, an enhanced permeability of the intestinal mucosa was witnessed by experimental findings in vivo and vitro. Our findings confirmed the previous study reporting that renal IR initiates a complex cascade of events that eventually result in intestine pathological changes and functional alterations [4].

Breakdown of the intestinal barrier would potentially lead to the translocation of bacteria and their products. In the setting of chronic uremia, intestinal barrier disruption associated endotoxinemia has been well documented [14, 15]. The similar processes in AKI have long been assumed [16]. In our study, a low grade level of endotoxinemia in rats of renal IR was detected, which was comparable to that in chronic uremia rats [15]. What calls for special attention here is that all procedures in our experiment were performed using sterile techniques. It seems plausible to assume that serum endotoxin may derive from the intestine other than surgical contamination, since the intestine contains the largest number of bacterial cells. In a mice model of renal IR, however, Diba Emal et al. failed to quantify detectable levels of endotoxin in the blood [17]. Similarly, the serum endotoxin levels of mice assessed 7 days after sham or IR were no difference in another study [18]. In contrast to the above-mentioned studies, rats were used in our study. As the loads of endotoxin and bacteria in the intestinal lumen may be quite different between animal species, the differences in the experimental animals (mice vs. rats) may result in different findings. In addition, factors such as the timing of endotoxin measurement and the sensitivity of endotoxin assay may also cause discrepancies among studies.

Despite the well-known detrimental role of endotoxin in the pathogenesis of sepsis AKI, it is not clear whether such a subclinical level of endotoxinemia in renal IR is sufficient enough to aggravate renal inflammation. Therefore, we next investigated the role of gut-derived endotoxin in kidney inflammation during renal IR. TLR4 is a pattern recognition receptor that serves as a endotoxin sensor, and whose activation recruits inflammatory factors and causes renal damage [19]. We demonstrated that, in a clinical related rat model of severe renal IR, the kidneys displayed an increased protein expression of TLR4. This was accompanied by a marked increase in renal proinflammatory mediators namely, TNF-a, IL-6 and MCP-1. Norfloxacin pretreatment reduced the grade of endotoxinemia in IR rats and this was followed by a reduction in TLR4 expression, IL-6 and MCP-1 production in the kidney. These findings suggest there might be a casual relationship between gut-derived endotoxin and renal inflammation during renal IR.

The degree to which gut-derived endotoxin actually contributes to renal injury is the questions we critically concerned. In our study, norfloxacin pretreatment produced no substantial benefits to renal outcome, in spite of its effects on alleviating renal inflammation. However, Diba and colleagues had reported that depletion of gut commensal bacteria with antibiotics reduced renal IR injury [17]. By contrast, germ-free mice were reported to have increased renal IR injury [20]. Thus, the role of intestine bacteria and its products in renal IR injury seems to be complicated and still needs to be clarified in future.

We observed that norfloxacin pretreatment prevented renal IR induced hepatic dysfunction. Similarly, TLR9 deficiency prevented liver damage secondary to severe renal IR but failed to reduce renal dysfunction and tubular necrosis [21]. These data and our findings suggest some unknown factors, which may be independent of kidneys, take part in the hepatic injury after severe renal IR. The liver interconnects with small intestines via portal vein and is the first organ of defense against gut-derived endotoxin. In normal conditions, small amounts of bacterial products enter the liver via portal vein and most of them are eliminated by Kupffer cells (KCs) [22]. When the flux of endotoxin overwhelms the phagocytotic capacity of KC, the endotoxin spills over into the systemic circulation [23]. Therefore, the liver is more susceptible to be assaulted by gut-derived endotoxin than the kidney. However, the role of gut-derived endotoxin in renal IR-induced liver injury remains to be investigated.

## Conclusions

Our results show for the first time that gut-derived endotoxin, resulting from an increased intestinal permeability after severe renal IR, subsequently amplifies intrarenal inflammatory response by activation renal TLR4 signaling. However, our results do not establish that antibiotic administration was effective in improving the overall renal outcome, in terms of histopathological changes and renal dysfunction. Even so, our intriguing findings may be the first step to understanding how to tailor therapies to mitigate intrarenal inflammation in select groups of patients, for example, those at high risk for renal IR.

## Additional file

Additional file 1: Uncropped Western blots: kidneys TLR4/GAPDH. (PDF 253 kb)

## Abbreviations

AKI: Acute kidney injury
ALAT: Alanine aminotransferase
ASAT: Aspartate aminotransferase
ELISA: Enzyme-linked immunosorbent assay
FD-4: FITC-Dextran
HE: Hematoxylin and eosin.
IL-6: Interleukin-6
IR: Ischemia reperfusion
LPS: Lipopolysaccharide
MCP-1: Monocyte chemotactic protein-1
MLN: Mesenteric lymph nodes
TLR4: Toll-like receptor 4
TLRs: Toll-like receptors
TNF-α: Tumour necrosis factor alpha
