# Supplementary material for: Gut derived-endotoxin contributes to inflammation in severe ischemic acute kidney injury
Source: BMC Nephrol. 2019 Jan 11;20:16. doi: 10.1186/s12882-018-1199-4 (PMC6329050; doi:10.1186/s12882-018-1199-4)
Supplement: Supplementary file 1 — Uncropped Western blots: kidneys TLR4/GAPDH. (PDF 253 kb) [file 12882_2018_1199_MOESM1_ESM.pdf]

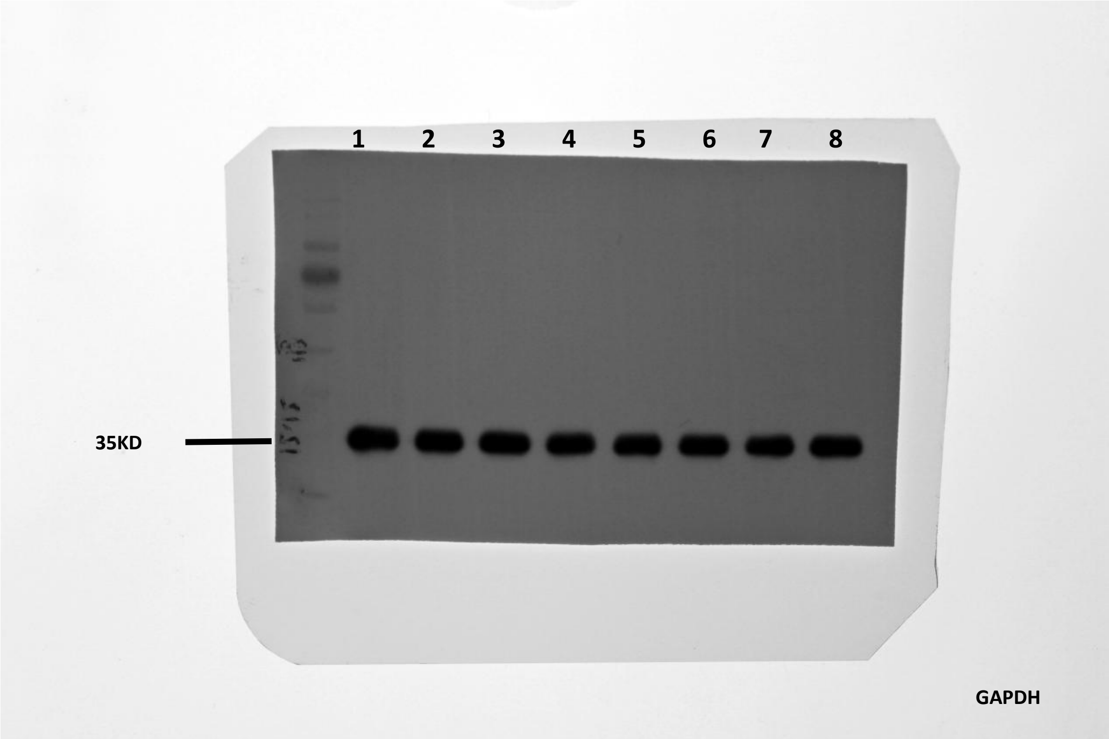
1 2 3 4 5 6 7 8
35KD
GAPDH

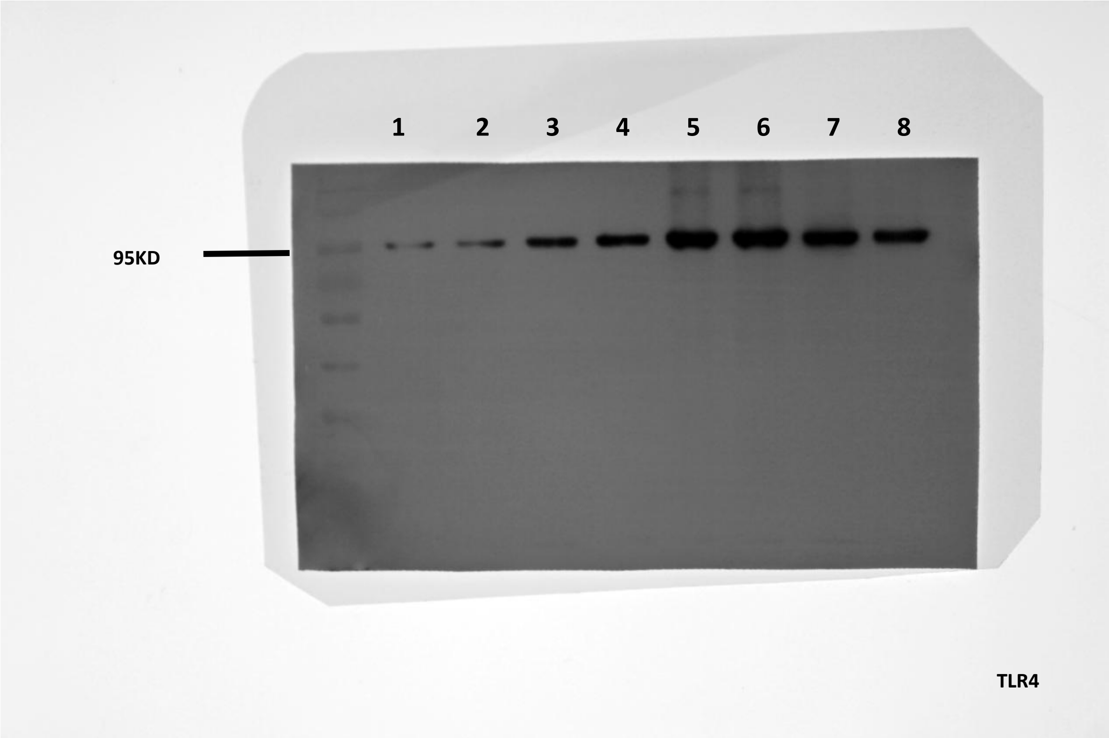
1 2 3 4 5 6 7 8
95KD
TLR4

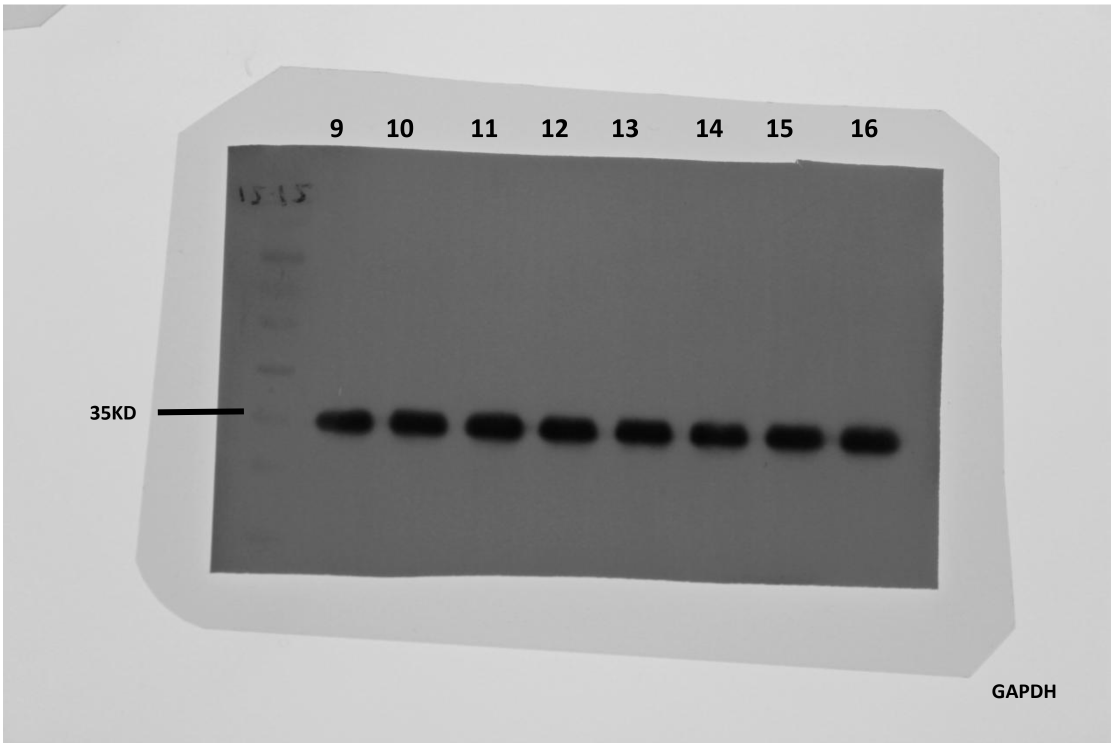
9 10 11 12 13 14 15 16
35KD
GAPDH

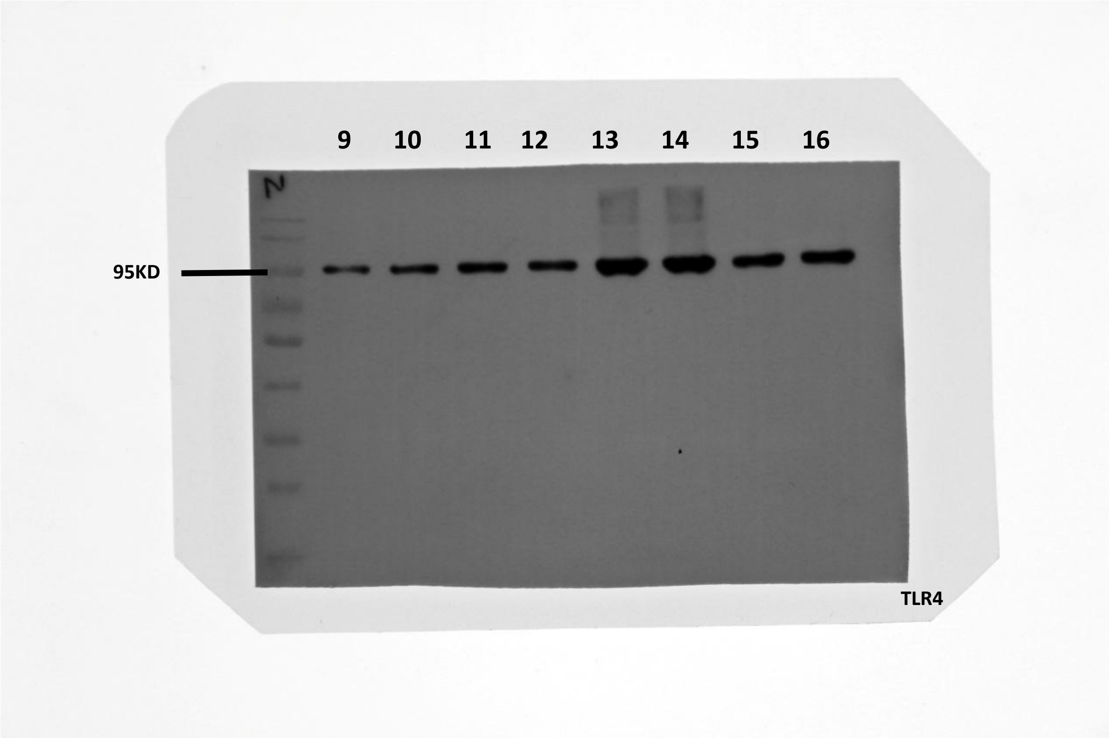
9 10 11 12 13 14 15 16
95KD
TLR4

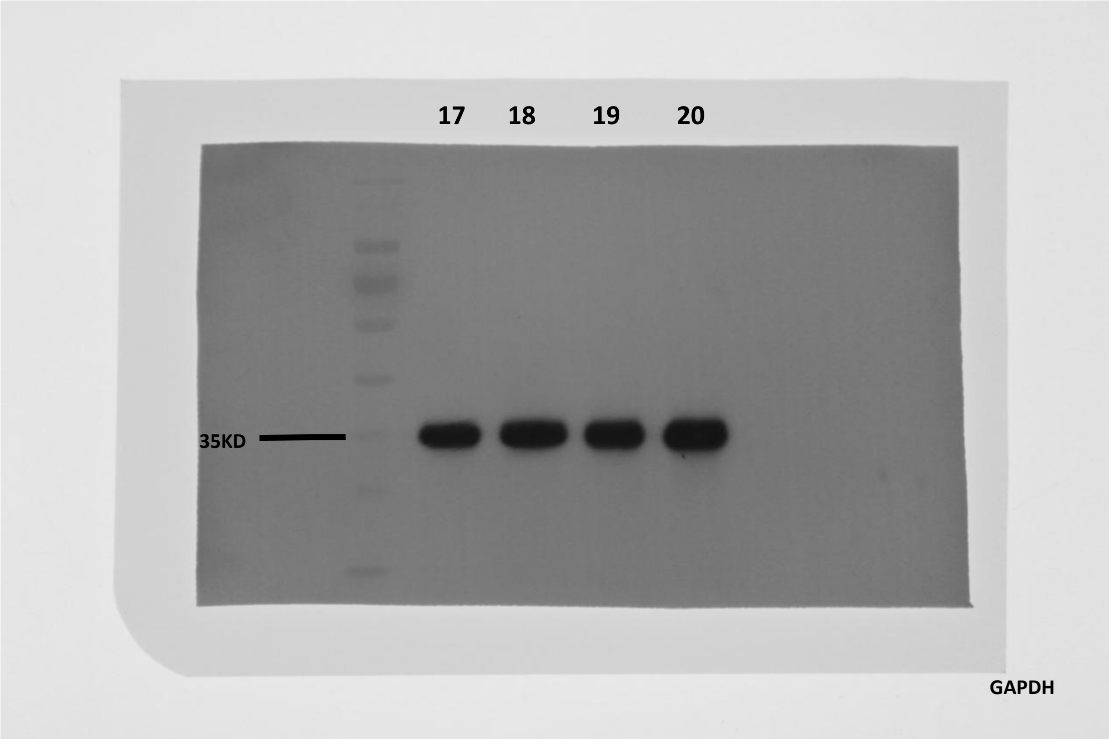

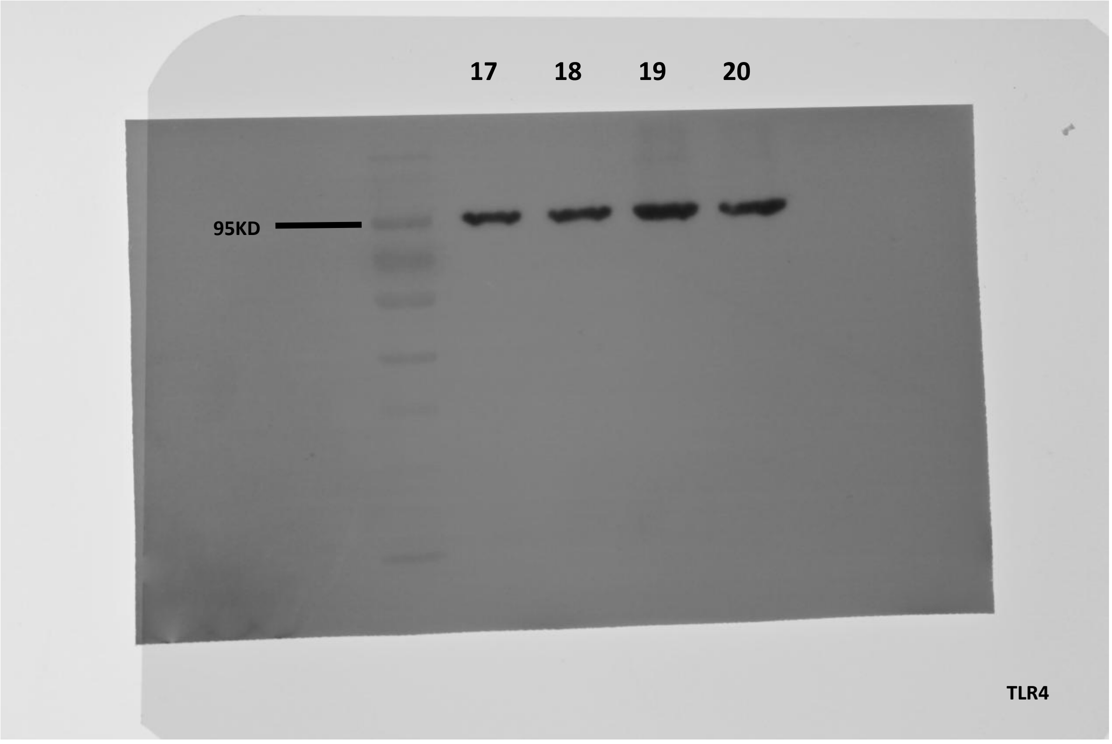

Uncropped Western blot images, these figures represent all blots that support results reported in Figure 3B. In these figures, full-length Western blot images are provided. Sham+Saline:1,2,9,10,17; Sham+Norflox:3,4,11,12,18;IR+Saline:5,6,13,14,19; IR+Norflox:,7,8,15,16,20.
